# Retraction: miR-19a protects cardiomyocytes from hypoxia/reoxygenation-induced apoptosis via PTEN/PI3K/p-Akt pathway

**DOI:** 10.1042/BSR-2017-0899_RET

**Published:** 2024-11-15

**Keywords:** apoptosis, cardiomyocyte, hypoxia/reoxygenation, miR-19a, PTEN/Akt pathway

This article “miR-19a protects cardiomyocytes from hypoxia/reoxygenation-induced apoptosis via PTEN/PI3K/p-Akt pathway” DOI: 10.1042/BSR20170899 is being retracted from *Bioscience Reports* at the request of the Editor-in-Chief and the Editorial Board. This follows receipt of a notification from a reader, alerting the Editorial Board to Figure 5C Control panel, part of which seems to appear as Figure 5C H/R+miR-19a panel. The authors were contacted regarding the concern raised and provided an explanation and a corrected image for Figure 5C H/R+miR-19a that addressed this issue. The authors additionally requested a correction to Figure 3B as they were uncertain if the published images were labelled correctly, which raised concerns regarding the handling and storage of the data in this figure.

The Editorial Office also requested the original Western blot data, which when compared to the article showed signs of modification in the published Western blot figures. Signs of digital editing were also identified in the provided original blots. Therefore the Editorial Office no longer has confidence in the validity of the results, and the Editorial Board stands by the decision to retract. The authors were asked to respond to the retraction, but did not agree or disagree.

